# Mézières Method as a practice of embodiment in patients with low back pain: a mixed study

**DOI:** 10.1080/07853890.2023.2265379

**Published:** 2023-10-17

**Authors:** Margareth Lorena Alfonso-Mora, Miriam Guerra-Balic, Ricardo Sánchez-Martín, Zandra Pedraza-Gómez, José Ramírez-Moreno, Adriana Lucía Castellanos-Garrido, Leidy Katerin Zambrano-Cristancho, María Leonor Rengifo Varona

**Affiliations:** a Universidad de La Sabana, Chía, Colombia; b Blanquerna School of Psychology, Educational Sciences and Sports, Ramon Llull University, Barcelona, Spain; c Universidad de Los Andes, Bogotá, Colombia; d International University of Catalonia, Barcelona, Spain; e Clínica Universidad de La Sabana, Chía, Colombia

**Keywords:** Low back pain, physiotherapy, Mézières Method, mixed study, practices of embodiment

## Abstract

**Introduction:**

The objectives of this study were to determine the effects of the Mézières Method (MM) on pain and disability related to low back pain (LBP), compared to a program of heat, massage and exercise, and to understand the meaning of the bodily experience with the MM.

**Patients and methods:**

Mixed methods convergent parallel design, combining an equivalent randomized clinical trial with a qualitative phenomenological approach. Sixty-one participants aged 18–65 years with chronic non-specific LBP lasting more than 3 months. Patients were randomized into two groups: the MM group (*n* = 29) and the comparison group (CG) who received heat, massage plus flexibility and strengthening exercises (*n* = 31). MM and CG participants underwent 10 one-hour physical therapy sessions over a 5-week period and were evaluated three times: pre-intervention, post-intervention and follow-up at 6 weeks after the end of treatment.

**Results:**

Both groups reported positive effects on LBP . MM group showed superior effects in pain relief in the short term (Cohen’s *D* 0.80; *p* = 0.004). Participants interpreted the interaction with the MM as a teaching–learning process that allowed body awareness.

**Conclusion:**

Both treatment were similarly beneficial but MM had superior effects on pain in the short term. MM is perceived by the participants as a teaching–learning process focused on body awareness that facilitate effective management of LBP.

## Introduction

Low back pain (LBP) is one of the three main causes of consultation with health services worldwide [1]. The 2019 Global Burden of Disease showed that LBP has remained, since 1990, the fourth highest cause of disability worldwide in the productive population. It has ascended two positions in the population aged 10–24 years since 1990, for 2019 is the fourth cause of consultation [2]. In physiotherapy, various treatment alternatives are performed, such as massage, manual therapy and exercise [3], education [4] and the application of thermal media [5], among others. Guidelines about LBP physiotherapeutic treatment are clear in promoting movement, education with body awareness (BA) and cognitive treatments could be a benefit for LBP patients. Current guidelines LBP contemplate different treatment options., Nevertheless, there is absence of considerations regarding body awareness [6].

This study investigates the Mézières Method (MM) as an intervention strategy for people with LBP that contemplate the person in integral view. MM is considered a practice of embodiment (PE) that promotes BA, including kinesthetic and proprioceptive sensations. It is proposed that from this awareness, it is possible to improve pain [7]. According to PE, BA focuses on the somaesthetic, understood as somatosensory activity, that includes proprioceptive, exteroceptive and interoceptive activity [8,9] as capacities of self-perception. PE seeks to enhance the acuity of the senses to achieve better health while releasing the tensions caused by bodily habits [9].

MM is a PE that develops BA by implementing intentional and individualized exercise, facilitated by the physiotherapist, and assisted by manual techniques and conscious breathing. The patient directs their attention to the points of the body that are compensating and tries to obtain better quality movement, which is proposed to translate into better balance, kinesthetics and proprioception [10]. Pepe et al. [11] suggest that MM also promotes the patient’s ability to manage their emotions, which improves physical and psychological well-being. Case study reports indicate that people treated with MM reverse their symptoms and experience improvements in BA [12,13].

The effectiveness of MM for LBP has been investigated by Lena et al. [14, 15] in three studies of athletes from different sports . MM proved more effective than exercise alone in all trials. In each study, a 24-week program was delivered. Treatments were twice per week, totalling 48 occurrences of care, which, from the perspective of health care costs, could be excessive. The objectives of this clinical trial were to determine the effects of MM on pain and disability in people with LBP, using a more relevant clinical protocol of a 5-week program. MM was compared to a program of heat, massage and exercises (control). We also wanted to understand the participants’ body experience when performing MM as to date there have been no mixed methods studies evaluating MM from both perspectives of pain relief and BA. The hypothesis was that MM would provide equal symptomatic relief to the control program. Secondly, MM would improve participants’ BA.

## Patients and methods

### Setting

This study was conducted at the University Clinic of La Sabana Colombia, a clinical centre that has high rates of consultation by patients with LBP.

### Design

A mixed method, convergent design was employed [16] with an equivalence design. The integration of the methodological procedures was through parallel collection of quantitative randomized control trial (RCT) and qualitative data from participants who received MM. The comparison group (CG) participated in the RCT only. Confirmation of the quantitative data was sought by seeking categories with explanatory citations that would expand the understanding of the statistical results We utilized a phenomenological approach [17]. Ethical approval was obtained from the Institutional ethics committee of Clinic of La Sabana Colombia. The trial followed the CONSORT guidelines and was registered in ClinicalTrials.gov (NCT03738306); it was developed since March of 2019 until March of 2020 (finished in anticipated way for COVID pandemic); all participants signed informed consent to study participation.

### Patients

**Figure 1.**
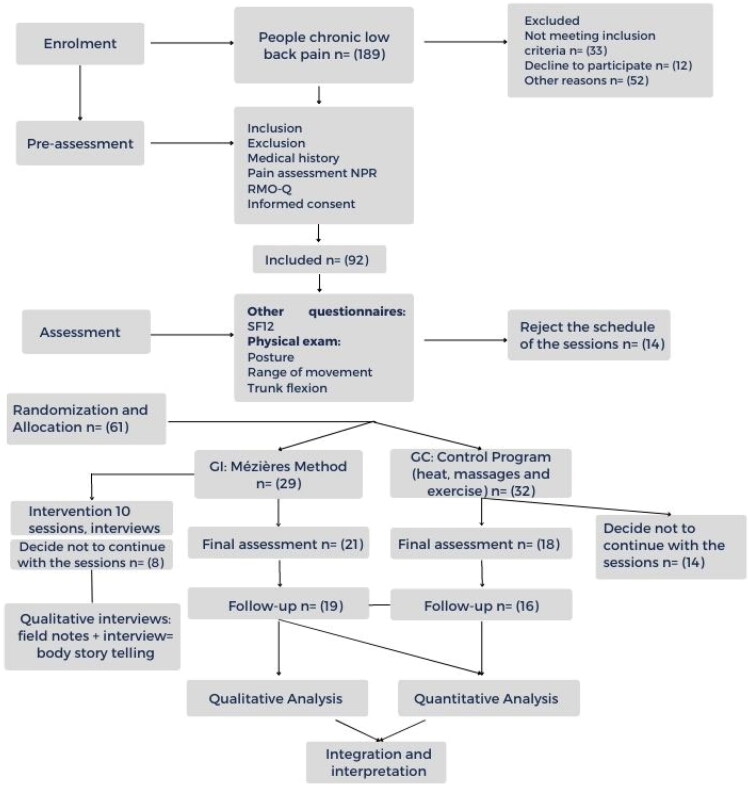
Flowchart of the study procedure.

Males or females aged 18–65 years with chronic non-specific LBP lasting more than 3 months. Reported disability was to score greater than 4/24 on the Roland Morris Questionnaire (RM) [18] and pain to score greater than 2/10 in the past 2 weeks on the Numerical Pain Rating (NPR) scale [19]. Exclusion criteria were disease associated with LBP (e.g. tumours), body mass index greater than 35, radiculopathy, congenital fusions, postsurgical segmental malalignment and high risk LBP with STarT Back Screening Tool (SBST) [20] (Figure 1). From 189 potential participants, 61 were included in the trial.

Sample size estimates were calculated based on expected effect size of 0.78 for the primary outcome (pain score). With this assumption, 27 participants per group were required to obtain 80% statistical power with a 5% significance level.

### Randomization

Group allocation in the randomization process was determined by computer-generated numbers (Microsoft Excel). Assignment was conducted by an independent researcher. The independent researcher assigned the participant to the group and contact details to the treating physiotherapists in a sealed envelope. Patients were contacted and informed about the schedules of each intervention group according to allocation made.

### Outcome measurements

Three measurements were performed to evaluate the variables of the study before and immediately after the intervention and at the 6-week post-treatment follow-up. They were performed by an independent researcher who was blind to the participants’ treatment allocation; questionnaires were assisted by a blinded researcher.

### Primary outcomes

Pain intensity was measured at each time point using NPR [19]. Disability was measured using RM [18,21]. The relevant clinical change of this scale is five points.

### Secondary outcomes

Quality of life (QOL) was evaluated with the Spanish version of the SF12 which consists of 12 questions related to physical and mental health. The total scores range from 0 to 100, where a score closer to 0 indicates the lowest level of health and 100 indicates the highest level of health [22].

**Figure 2.**
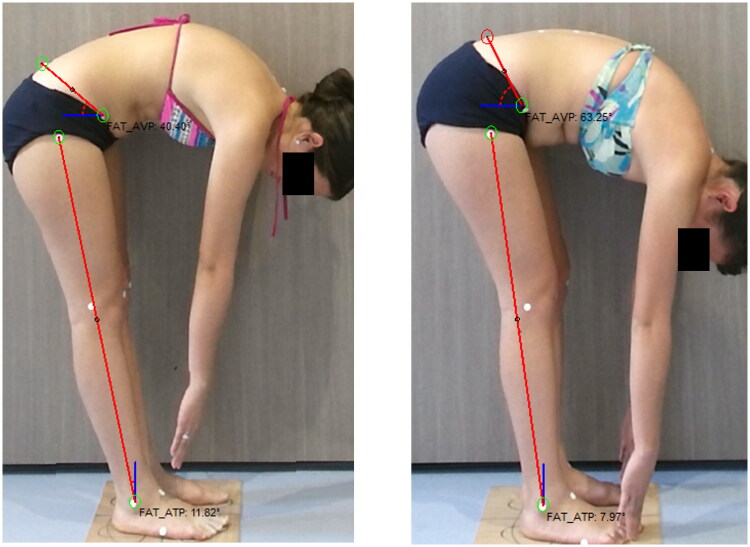
Changes in flexibility.

Posture and flexibility were evaluated with the ADiBAS^®^ system (PhysicalTech, Barcelona, Spain) [23]. Posture was assessed in standing and flexibility was assessed in a trunk flexion test. Variables for posture were thoracic kyphosis (TK) and lumbar lordosis (LL); TK and LL are a 3D reconstruction from posterior plane of spine; TK is an angle of the thoracic curve between T1 and T12 and LL is an angle of the lumbar-sacral curve between T12 and S2 [7]. Regarding flexibility, anterior pelvic tilt (APT) and posterior translation of the legs (PTL) were assessed. The APT during trunk flexion expresses the extensibility of posterior hip and leg muscles and it is measured by the angle of a line between the anterior superior and posterior superior iliac spines to the horizontal (Figure 2). PTL is the angle estimating the posterior translation of the tibia in relation to a vertical line from the lateral malleolus. The procedure is fully described in the research protocol [7].

### Interviews

Qualitative data from the MM intervention was obtained in sessions 4, 8 and 10 by means of a semi-structured interview that inquired on three aspects: (1) body sensations with the MM before and after session; (2) changes in the LBP and (3) perception of the treatment received overall. The interviews were conducted in a different setting than the MM sessions; the duration was 20–60 min; it was developed by a MLAM, who applied MM intervention; interviewer did not have previous relationship with the participants. Interviews were recorded and transcribed for analysis. Details are provided in the study protocol [7]. Data collection were made based on obtaining a diverse sampling and reaching data analysis saturation. When no new categories emerged from the data, the team stopped data collection, supported on that 20 interviews were included from MM group.

#### Intervention

All participants received two treatment sessions per week for 5 weeks.

The MM intervention was performed by a physiotherapist certified in MM. She was blind to the randomization and evaluation processes. The intervention was personalized with a duration of 45–60 min per session.

Global stretches related to the symptoms of LBP were included, with an emphasis on a region of the body in each session. Sessions 6–10 were combined according to the needs identified in the first five sessions [24]. A description of the program is presented in the Supplemental data.

In CG the protocol was based on a proposal by Aluko et al. [25] and Carpes et al. [26] and included thermal modalities [27], massage [28] and muscle stretching and strengthening. Each session lasted for 30–45 min. The procedure is fully described in the research protocol [7]. CG intervention was performed by an independent physiotherapist trained in the protocol.

#### Data analysis

The statistical package SPSS IBM version 21 was used for data analysis. Analysis included descriptive statistics. Normality was checked with Kolmogorov–Smirnov. An intention-to-treat analysis was performed using a repeated measures ANOVA. If the assumptions of homoscedasticity and equality of variance were met, we included the baseline variables that presented differences between groups as covariates. If not met, nonparametric analysis of variance (Kruskal–Wallis test) for between-group comparisons at the three evaluation times for the pain, disability, QOL, flexibility and posture variables was included. Between-group differences for each measure were explored using either Student’s *t* test for independent samples or the Mann–Whitney *U*-test when the variables presented a non-normal distribution. The Friedman test was used to test for the intragroup changes at the three measurement times. Effect size was calculated with Cohen’s *D* between-group comparison, an important effect was considered with scores higher than 0.70.

The analysis of the data of the qualitative study began with manual transcriptions of the interviews. Names were coded with initials and the consecutive number of the interview to protect the identity of the participants.

The formulation of meanings was developed, beginning with the reading of each transcript several times. This process was carried out by the principal investigator and four final year physiotherapy students. After reading the first interview, a meeting was established to create the initial codes. Each researcher coded an additional interview to validate the interpretation which was evaluated in another group meeting. The codebook for data analysis was created using the emergent codes from an interview. Similar codes were grouped into categories that were continuously refined and organized. After axial coding, the relationship between the categories was sought, thus establishing the subcategories. Only those that approached the identified central category were considered. The software used for the qualitative analysis was ATLAS.ti.

Integration of the results of the RCT and the qualitative study was developed in a comparative way based on the data; the results are shown in the integration process combining each variable with the categories emerged by interviews [29].

## Results

Sixty-one participants with LBP entered the study, with an average age of 37 ± 7 years. Most participants reported not taking medications and the average history of pain in both groups was close to 6 years. There were no between-group differences in demographic characteristics of the participants in baseline measurements (Table 1).

**Table 1.** Participant characteristics and baseline data.

	MM				
Variable	%		Control intervention		*p* value
Gender	M	31	M	25	0.40
	F	69	F	75	
Taking medications	Yes	24	Yes	34	0.27
	No	76	No	66	
Variable	MM		Control intervention		*p* value
	Mean (SD)				
Age (years)	39.5 (13.8)		35.2 (13.4)		0.22
Time with pain (years)	6.0 (6.8)		5.6 (5.3)		0.25
BMI	24.9 (3.4)		24.3 (3.5)		0.8
Pain NRS 0–10	6.5 (1.9)		6.2 (1.1)		0.20
Disability RMD 0–24	9 (5.2)		9 (3.8)		0.95
Physical health SF12	50.98 (7.2)		47.65 (7.6)		0.12
Mental health SF12	49.98 (9.5)		48.97 (8.4)		0.65

The qualitative data grouped into four subthemes: pain relief, activities of daily living (ADL), QOL, balance and release of tension. Each subtheme was related to a quantitative outcome.

### Pain

Pain scores within each group showed a clinically relevant and statistically significant reduction post-treatment and improvements were maintained at follow-up (Table 2). The between-group analysis revealed significant differences with clinical relevance between pain scores post-intervention in favour of the MM (effect size 0.80). However, at the 6-week post-treatment follow-up, there were no differences between groups (Table 2).

**Table 2.** Results between group comparisons for pain, disability and QOL measures.

	CG			MM			Difference between groups 5 weeks *p* value	Difference between groups Follow-up *p* value	Size effect Cohen’s *D*
	Pre	5 weeks	Follow-up	Pre	5 weeks	Follow-up			
NPR	6.2	3.3*	2.0*	6.5	2.05*	2.7*	***1.3 (0.04)***	0.7 (0.96)	***0.811***
RMDQ	9.0	3.0*	2.0*	9.0	2.00*	1.0*	1.0 (0.11)	1.0 (0.36)	0.383
QOL Physical	47.7	47.6	48.55	51.0	51.9	46.5*	4.9 (0.06)	1.9 (0.69)	0.553
QOL mental	49	46.88	43.85	50	50.5	47.6	3.6 (0.22)	3.8 (0.15)	0.273

*Friedman test, within-group significant difference.

RMDQ: Roland Morris Disability Questionnaire.

Qualitatively, pain was expressed in the subtheme pain relief and was reported by all MM (20 interviewed) participants. Accordingly, the qualitative data were consistent with the numerical data. Pain was not eliminated, but its reduction produced a lower rating. Participants reported the following:

I had been feeling pain in a more continuous way. After the sessions it does not persist as much, it disappears a little, it comes back again, it appears and then disappears. (GM)

Well, for me, it was completely satisfactory because I no longer have the pain, it is more when the pressure is done and at a specific point. (LC)

### Disability

Analysis revealed statistically significant and clinically relevant changes (difference >5; *p* < 0.005) in Roland Morris Disability Questionarie (RMD) scores within each group post-intervention and improvements were maintained at the 6-week follow-up. There was no difference in the improvement of disability between groups (Table 2). The difference for both groups in this variable was clinically important.

Qualitatively, this variable relates to two subthemes: pain relief and ADL because participants not only referred to pain relief but also related it to improvements in their ADL. For these categories, the number of citations listed in the pain relief code was maintained. They reported the following:

It’s easier to get up, and I don’t feel that pain that I felt before, that my back was going to burst when I got up. I was getting used to that pain; now, I am used to not feeling it, and when I feel it, it’s like ‘I am doing something wrong’ … (AROM)

Hey, I have not had the episodes of feeling stuck that are the most frightening, not being able to drive a car, or walk, as happened to me before, or feeling that you cannot even turn your neck because you are stuck. I have not had those episodes. (CO)

### QOL

The analysis of QOL scores revealed that there were minor improvements in the physical component within the MM group and for mental health in the CG. However, there were no significant differences between groups at any follow-up point (Table 2).

Qualitatively, 20% of the MM participants referred to the term QOL during the interviews, and some perceived changes.

The change in pain […] is quality of life because one already has certain tools to control pain. I no longer need to reach the point of no return […] at least, I know how to manage it, and I know how to have quality of life, […] Knowing that if the pain is going to start, I do what you taught me, breathing, and that it is working for me. It has allowed me to be well. I am calm and I have a good quality of life. (JAC)

When I was hurting, the pain was in my waist, my spine. It was terrible. I could not move. My quality of life ended. (JOMU)

### Posture

The analysis revealed no changes in posture in either of the two groups (Table 3). However, qualitatively, MM participants reported that they could adopt postures that did not cause them pain, and they had less fatigue. Measures of posture did not coincide with those bodily sensations reported by the participants.

**Table 3.** Results of the between group comparisons for posture and flexibility.

	CG			MM			Difference between groups 5 weeks *p* value	Difference between groups Follow-up *p* value	Size effect Cohen’s *D*
Posture	Pre	5 weeks	Follow-up	Pre	5 weeks	Follow-up			
TK	40.30	37.4	39.6	33.20	29.5*	32.4	7.9 (0.010**)	7.2 (0.11)	0.869
Lordosis	46.20	49.1	46	40.00	37.4*	41.9	**11.7 (0.007** ** **)**	4.1 (0.17)	0.937
Flexibility									
APT	56.3	58.1	63.5	54.2	64.7*	62.9*	6.6 (0.257)	0.6 (0.31)	0.344
PTL	9.4	8.2	14.7	10	8.2*	7.9*	0 (0.443)	***6.8 (0.001)***	***0.798***

*Friedman test within-group significant difference.

**One-way ANOVA between-group significance difference.

Qualitatively, posture matched the *balance* subtheme and was reported in 95% of the interviews. This subcategory is described as a harmonious body position, which avoids the positions of the body that generate fatigue and therefore minimizes energy expenditure and ultimately generates comfort.

I also feel like a change in posture, especially at the cervical and dorsal level, improved postural alignment. I now have a lot of awareness of posture, […]. (LC)

### Flexibility

There were significant changes for the flexibility variables for the MM both in APT and posterior pelvic tilt (PTL; Figure 2). APT changed 10 degrees (*p* = 0.019), and PTL changed 3 degrees (*p* = 0.009) in the MM group while in GC it was 2 degrees (*p* = 0.29) and 1.2 degrees (*p* = 0.30) respectively (Table 3).

Qualitatively, the subthemes *release tension* and *more flexibility* were reflective of flexibility. These are understood as the ability of the person to release the tension in his or her muscles that may be related to poor flexibility. There were 93 citations in this regard, expressed by 50% of the MM participants:

Pain has decreased, and I have felt more flexibility, I have also felt as if I could stretch more. (GM)

… Also, when I bathe, when I apply the cream, I could not bend to put cream on my legs because I felt a lot of pain. I felt stiff. I did not feel capable of bending […] now I feel more flexible. (MC)

The subthemes of releasing tension and greater flexibility did not present a degree of saturation as occurred with pain reduction and improvement in balance, the participants could refer to greater flexibility through codes, such as releasing tension.

## Discussion

This trial is the first to use a mixed method design to evaluate the effects of MM from perspectives of pain, disability and QOL. The equivalency of the results of the two interventions in this study supports the benefits of MM as an alternative physiotherapeutic treatment for people suffering from LBP.

The improvements in pain were greater in the MM than the CG immediately after treatment but both groups achieved similar pain relief at the 6-week follow-up. Thus, our results are in agreement with those of Lena et al. [14] and Payares et al. [18] in the studies on athletes, and importantly, we were able to demonstrate benefit after a 5-week intervention program.

Participants receiving both the MM and CG similarly reported reduced RMD post-intervention, and improvements were maintained at the 6-week follow-up as found by Lena et al. [12,14,30], at 12- and 24-week measurement occasions. Neither the MM or the CG had significant effects on QOL, again similar to findings of Lena et al. [12]. The lack of effect of MM is consistent with the findings of Wen et al. [31] who found that mindfulness interventions, as a PE similar to MM, do not generate an important effect in QOL in LBP patients, but the participants acquired relevant benefits in pain and disability [32]. A systematic review found that QOL improved with moderate to high intensity exercise interventions. The exercise interventions in both groups were low to moderate, which might explain the lack of effect on QOL.

Our study also showed positive and superior effects of MM in flexibility specifically APT. Lena et al. [14] also found significant changes in flexibility between groups at 24 weeks [12,14,30]. Prior studies have not considered posture as an outcome even though postural awareness is a key component of MM. Nevertheless, neither MM or the CG resulted in changes in posture measures, despite MM participants reporting changes in sensations of functional postures rather than the static upright posture as measured in this study.

Our CG also generated positive effects on LBP. This was not surprising as systematic reviews and clinical practice guidelines support the use of exercises for the management of LBP [33–35]. We also included multimodal treatment program as there is some evidence that physical agents in conjunction with exercise can provide some additional benefits in terms of pain relief [27,36–39].

The findings of the qualitative study were around the main theme of a teaching–learning process that promotes BA. Subthemes that may explain the body experiences with MM were pain relief, ADL, QOL, balance and release of tension. Our findings suggest that MM may generate a better health outcome in LBP patients by using sensitive postures and exercises that promote voluntary control, other physiotherapeutic interventions promoting BA as Norwegian psychomotor physiotherapy [40] and Basic Body Awareness Therapy [41]. They further recognize that the physiotherapeutic process has a body pedagogical function that must be strengthened and structured from the embodiment. There is consensus that whatever the method, increasing knowledge and BA contribute to pain relief and patients’ ability to better use non-provocative movements and postures [13,41–44].

This study has limitations. The major one is the high dropout rate which occurred because the trial was interrupted by the COVID-19 pandemic. This caused several participants to be unable to continue with the study. Another limitation is that a qualitative approach was not used for the CG group.

## Conclusion

The MM relieved pain more effectively than the control intervention of heat, massage, strengthening and stretching exercises immediately after 5 weeks of treatment although both interventions were equally as effective at the 6-week follow-up time point, both interventions have shown results with clinical relevance in pain and disability. MM is perceived by participants as a teaching–learning process focused on BA that allows effective management of symptoms related to LBP. Based on the results of the study, MM can be recommended as a management option for patients with LBP as outcomes are equivalent to those obtained with exercise interventions as advocated in clinical practice guidelines.

## Supplementary Material

Supplemental Material

## Data Availability

The data availability is in the process of being published in the intellectum repository. If this information is required, please contact the author of the correspondence.
