# Supplementary material for: Mézières Method as a practice of embodiment in patients with low back pain: a mixed study
Source: Ann Med. 2023 Oct 17;55(2):2265379. doi: 10.1080/07853890.2023.2265379 (PMC10583626; doi:10.1080/07853890.2023.2265379)
Supplement: Supplemental Material [file IANN_A_2265379_SM0760.docx]

Appendix 1

Intervention Mézières Method

The order of interventions for each session was as follows:

| Session 1: Body positioning awareness exercises are performed both in the bipedal position and in the supine position. In the supine, one becomes aware of breathing, pelvic mobility, and lordosis. Repositioning of the body is performed seeking an alignment and the session ends with awareness of the position of the neck and if there is tension in any region, it is released. | Body awareness  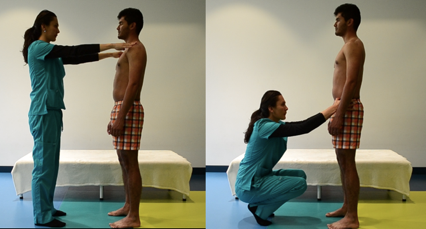  Breathing  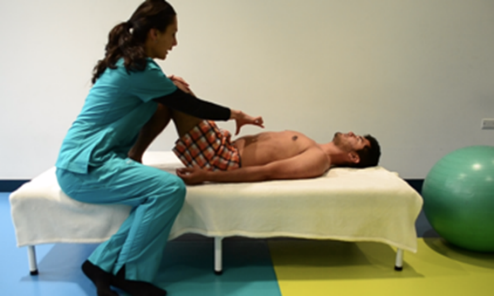 |
| --- | --- |
| Session 2: an asymmetric posture is performed for the posterior muscle chain, the posterior muscles are stretched to flex the coxofemoral mobility, the position is maintained with each leg for 10 to 15 minutes, while maintaining alignment of the trunk and head, the therapist helps, by means of verbal and tactile communication to identify which areas of the body require alignment, myofascial release of areas with evident tension is also performed. | 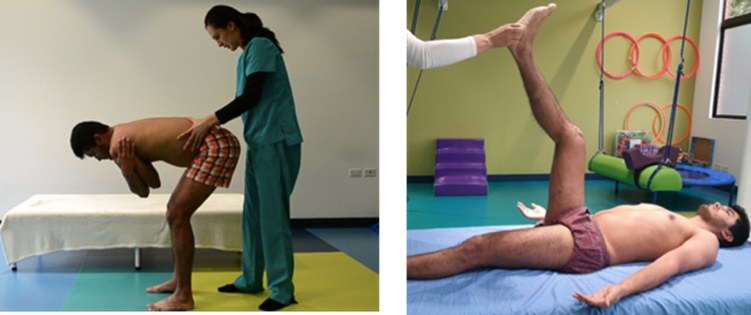 |
| Session 3: If the person tolerates the position, the supine symmetrical posterior chain stretch is performed, as in Figure 3 but with both legs, it is maintained for 20 to 30 minutes according to the tolerance of the person with rest intervals as needed. the need. If the person does not tolerate the symmetrical position, the asymmetric position of session 2 is performed again. |  |
| Session 4: bipedal posterior muscular chain posture. It is performed gradually, seeking the progressive descent of the trunk with a fixed point in the hip joint, keeping the back straight. Support of flexion of the knees was performed to release tension of the posterior chain, according to the need of the person. The tolerance position was maintained between 15 to 30 minutes, performing a deeper trunk flexion with each attempt. | 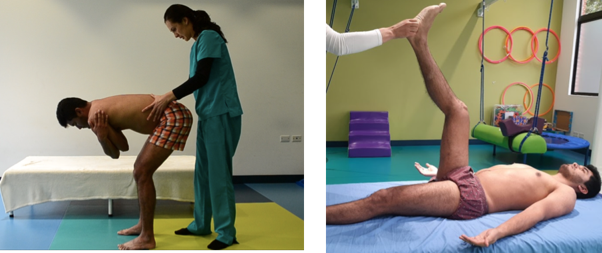 |
| Session 5: Thoracic-brachial muscle chain stretching was performed combined with stretching of the posterior muscle chain with symmetrical or asymmetric position as needed. | 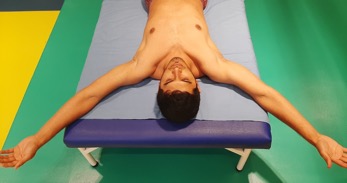 |
| In sessions 6 to 10, the positions that represented the most tension for the person were chosen, seeking the release of the muscle chain with the greatest impact on the lumbopelvic complex. During all sessions, free and rhythmic breathing was promoted without involving the accessory muscles. Exercises to improve the extensibility of the myofascial chains were performed, with the positions shown, avoiding local or distance compensations, seeking postural alignment and the activation of proprioception for a better sensory-motor integration during each phase of the session. Active readjustments were made according to the feedback of the therapist and the body, kinesthetic and tactile dialogue with him. For this, neuromuscular techniques were used (contraction-relaxation, contraction-inhibition), myofascial release techniques, deep massages on the muscular system, in order to improve the gliding planes of the myofascial chains, and thus, release tensions that would affect the lumbo-pelvic complex. These techniques were performed on each person respecting their pain thresholds. | |
